# Measuring cell surface area and deformability of individual human red blood cells over blood storage using quantitative phase imaging

**DOI:** 10.1038/srep34257

**Published:** 2016-10-04

**Authors:** HyunJoo Park, SangYun Lee, Misuk Ji, Kyoohyun Kim, YongHak Son, Seongsoo Jang, YongKeun Park

**Affiliations:** 1 Department of Physics, Korea Advanced Institute of Science and Technology, Daejeon 34141, Republic of Korea; 2 KI for Health Science and Technology (KIHST), KAIST Daejeon 34141, Republic of Korea; 3 Department of Laboratory Medicine, Eulji University Hospital, Daejeon 35233, Republic of Korea; 4 Department of Laboratory Medicine, University of Ulsan, College of Medicine and Asan Medical Center, Seoul 05535, Republic of Korea; 5 TOMOCUBE, Daejeon 34051, Republic of Korea

## Abstract

The functionality and viability of stored human red blood cells (RBCs) is an important clinical issue in transfusions. To systematically investigate changes in stored whole blood, the hematological properties of individual RBCs were quantified in blood samples stored for various periods with and without a preservation solution called citrate phosphate dextrose adenine-1 (CPDA-1). With 3-D quantitative phase imaging techniques, the optical measurements for 3-D refractive index (RI) distributions and membrane fluctuations were done at the individual cell level. From the optical measurements, the morphological (volume, surface area and sphericity), biochemical (hemoglobin content and concentration), and mechanical parameters (dynamic membrane fluctuation) were simultaneously quantified to investigate the functionalities and progressive alterations of stored RBCs. Our results show that stored RBCs without CPDA-1 had a dramatic morphological transformation from discocytes to spherocytes within two weeks which was accompanied by significant decreases in cell deformability and cell surface area, and increases in sphericity. However, the stored RBCs with CPDA-1 maintained their morphology and deformability for up to 6 weeks.

Blood storage is important for emergency transfusions during surgery and the treatment of anemia. According to current protocols, human red blood cells (RBCs) can be stored up to 42 days at 1–6 °C in a preservation solution^1^. This solution, containing citrate, phosphate, dextrose, and adenine (CPDA-1), prevents blood coagulation by chelating calcium and supplies the nutrient glucose and adenosine triphosphate (ATP) while maintaining a low pH. CPDA-1 is commonly used in blood banking and has been known to maintain the functionality and viability of stored RBCs for up to 6 weeks.

Because of the clinical importance of blood transfusions, one of the important issues is whether long storage times of banked blood affect the properties of the blood components. Several *in-vitro* studies have been done to address permanent alterations in RBC properties using diverse experimental techniques. Hematological tests showed noticeable increases in red cell distribution width (RDW), while other parameters including the mean corpuscular volume, mean corpuscular hemoglobin and mean corpuscular hemoglobin concentration did not change significantly^2^. In addition, the rate of morphological changes from discocyte to spherocyte gradually increases and becomes 50% at two weeks^3^. The relative biochemical parameters including 2,3-DPG, potassium, pH, and ATP and nitric oxide levels are also changed toward unfavorable conditions for RBC viability^4^, which is related to the functionalities in ion transporters in RBCs^5^,^6^,^7^. Elongation index tests^3^,^8^, optical tweezers techniques^9^,^10^, and ektacytometry experiments^11^,^12^,^13^ have revealed that stored RBCs lose deformability as a function of the storage period. Recent studies have reported a decrease in the dynamic membrane fluctuations of stored RBCs^14^ and a delay in passing through a narrow microfluidic channel as a result of large cell deformations^8^ over time in storage.

Despite *in-vitro* experiments reporting alterations in RBC properties, connections between the patient risk in blood transfusion and the storage period of the banked blood are unclear. It has been reported that progressive deteriorations in the functionalities of RBCs during blood banking/storage were associated with the mortality of transfused patients^15^,^16^,^17^. Therefore, in clinical practice, Koch *et al*. recommended to use RBCs stored less than 14 days due to an increase in the risk of death and complications although the shelf life of stored RBCs is much longer^18^. However, recent clinical studies have contradicted the adverse effects of long stored blood because no dependence was observed for patient mortality on the stored period of the transfused blood^19^,^20^.

Although previous studies on stored RBCs have significantly enhanced our understanding of storage lesions, none of them were able to investigate the morphological, biochemical, and mechanical parameters of individual RBCs simultaneously. Moreover, many of the previous methods only measured RBCs in the stored blood with CDPA-1 and thus are not well suited to investigate the effects of the preservation solution on the properties of individual RBCs.

Here, we systematically investigated the alterations of biophysical properties of individual RBCs as a function of the storage duration up to 6 weeks. With 3-D quantitative phase imaging (QPI) techniques and 3-D refractive index (RI) tomograms, the dynamic membrane fluctuations of individual RBCs were simultaneously and quantitatively measured based on the principles of laser interferometry and optical diffraction tomography^21^,^22^. From the optical measurements, the morphological (volume, surface area and sphericity), biochemical (hemoglobin content and concentration), and mechanical parameters (dynamic membrane fluctuation) of individual RBCs were simultaneously quantified to investigate the functionalities and their progressive alterations in stored RBCs. The measurements were done every few days up to 6 weeks to investigate the alterations of these RBC parameters systematically over time in storage. To investigate the effects of the preservation solution on the RBC parameters, we tracked two groups of RBCs: one stored in the absence and the other in the presence of a preservation solution known as CPDA-1. Our results show that the rate of morphological transformation from discocytes to spherocytes increased as the storage time increased, which was accompanied with increased membrane stiffness. For the stored RBCs without CPDA-1, all the RBCs became spherical shape within a week, whereas the storage agent CPDA-1 prevented the morphological transformation of the RBCs and maintained a healthy level of dynamic membrane fluctuations in the RBCs for up to 6 weeks in storage.

## Results

### Morphological alterations in RBCs over time in storage

To study how the morphology of the stored RBCs changes over time in the absence and presence of the preservation solution CPDA-1, 3-D RI tomograms of individual stored RBCs were measured with common-path diffraction optical tomography (cDOT) at days 1, 5, 13, 20, 27, 34 and 41 of the storage period. The RBCs were stored at 4 °C. cDOT is a highly sensitive 3-D QPI technique which is capable of measuring both the 3-D RI tomogram and dynamic membrane fluctuation of individual cells (see Materials and Methods)^23^.

**Figure 1:**
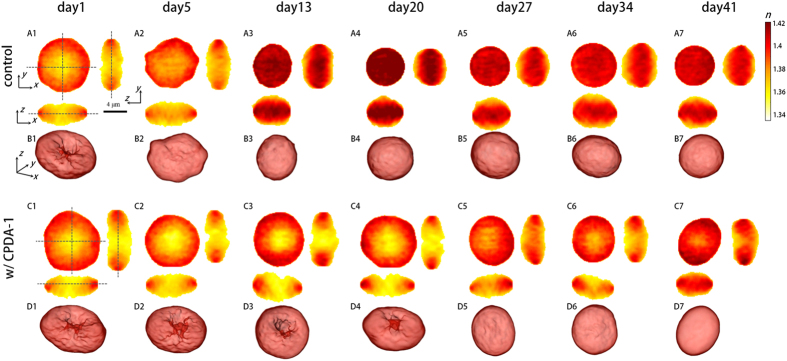
Cross-sectional slices of reconstructed RI tomograms of stored RBCs without CPDA-1 as the control (A1–A7) and with CPDA-1 (C1–C7) at days 1, 5, 13, 20, 27, 34, and 41 of the storage period, respectively, in the *x*-*y* (left), *y*-*z* (right), and *x*-*z* (below) plane (B1–B7 and D1–D7). Corresponding rendered isosurfaces of 3-D RI maps for the control and CPDA-1, respectively (*n* > 1.360).

Representative RI tomograms are shown in Fig. 1. The measured 3-D RI tomograms of the RBCs without CPDA-1 showed significant morphological changes over time in storage (Fig. 1A,B). The RBCs underwent morphological transitions from discocytes to echinocytes on day 5 of storage. Specular structures emerged in the RBC membranes, and their dimple structures in the center disappeared. After two weeks, all the RBCs without CPDA-1 became spherocytes, that is, the RBCs had spherical shapes.

In contrast, characteristic thin, doughnut-like shapes in the discocytes were still found in the RBCs stored with CPDA-1 up to 3 weeks of storage (Fig. 1C,D). After 20 days of storage, the portion of spherocytes increased among the RBCs stored with CPDA-1. These morphological changes for both RBC groups over long storage periods were consistent with a previous report in which scanning electron microscopy was used^3^. These morphological transitions can also be clearly seen in the rendered isosurface images of the reconstructed RI tomograms (Fig. 1B,D).

**Figure 2:**
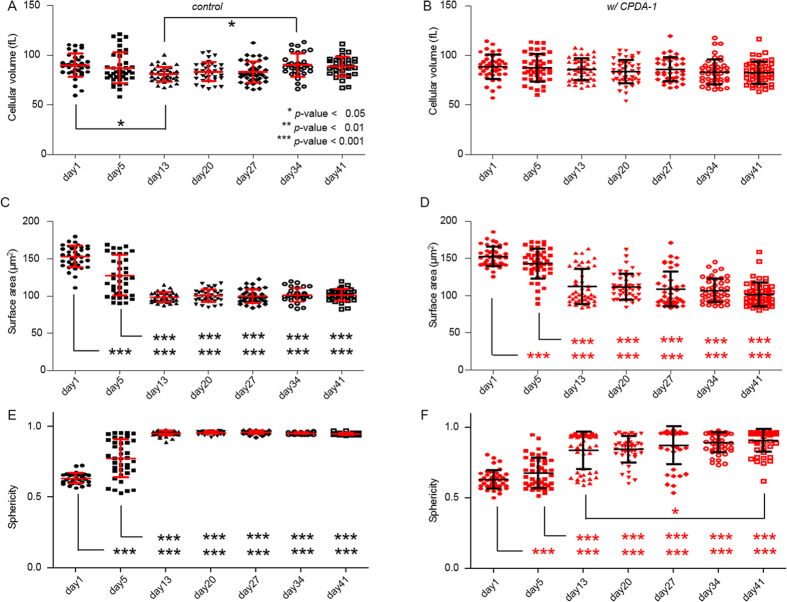
Morphological red cell indices of the stored RBCs without CPDA-1 as the control (**A,C,E**) and with CPDA-1 (**B,D,F**) at days 1, 5, 13, 20, 27, 34 and 41 of the storage period: (**A,B**) Cell volumes, (**C,D**) cell surface areas, (**E,F**) sphericities. Each symbol corresponds to an individual RBC measurement. The horizontal solid line is the mean value with an error bar showing the standard deviation.

For a quantitative analysis, the morphological parameters including the cell volume, cell surface area, and sphericity were calculated from the measured 3-D RI tomograms (see Materials and Methods). The results are shown in Fig. 2. During the 41 days of the storage period, the volumes of the RBCs were maintained for both the control and the RBCs with CDPA-1 (Fig. 2A,B). The mean values for the cellular volumes of the stored RBCs without CPDA-1 at days 1, 5, 13, 20, 27, 34, and 41 of the storage period were 89.7 ± 11.9 (n = 37), 87.0 ± 15.6 (n = 39), 80.7 ± 7.4 (n = 39), 83.2 ± 9.8 (n = 36), 82.8 ± 10.6 (n = 39), 89.9 ± 11.6 (n = 32), and 88.4 ± 9.9 fL (n = 34), and with CPDA-1 88.4 ± 12.2 (n = 43), 87.2 ± 13.9 (n = 44), 85.9 ± 11.0 (n = 47), 83.3 ± 11.9 (n = 45), 86.0 ± 12.0 (n = 37), 83.2 ± 12.6 (n = 43), and 82.5 ± 11.1 fL (n = 49), respectively. The unchanged cellular volume in the RBCs stored with CPDA-1 is consistent with a previous study using a complete blood machine, in which a large number of RBCs were averaged^2^.

The surface areas of the RBCs stored without CPDA-1 started to decrease from day 5, and almost all RBCs began to have similar small sizes after 13 days of storage (Fig. 2C). In contrast, the surface areas of the RBCs stored with CPDA-1 started to decrease gradually over a long time exhibiting large cell-to-cell variations. The mean values for the surface areas of RBCs without CPDA-1 at the corresponding storage periods were 152.7 ± 14.7, 127.7 ± 27.8, 98.5 ± 6.6, 100.2 ± 8.5, 99.5 ± 9.0, 101.6 ± 9.1, and 101.0 ± 8.0 μm^2^, respectively. At day 5, the surface areas decreased by 16% and further decreased by 33% on day 13. The mean values for the surface areas of the RBCs with CPDA-1 at the corresponding storage periods were 152.4 ± 13.3, 142.8 ± 20.1, 112.0 ± 23.5, 111.7 ± 17.2, 108.8 ± 23.7, 106.8 ± 15.7, and 101.5 ± 16.0 μm^2^, respectively. At days 5 and 13, the surface area decreased by 6 and 26%, respectively. After day 13, the standard deviations for the cell surfaces area of the RBCs stored with CPDA-1 were three times larger than those for the control RBCs.

To quantitatively analyze the morphological transition from healthy discocytes to abnormal spherocytes, the sphericity was calculated from the measured cell volume and surface area (see Materials and Methods). A sphericity of 1 corresponds to a perfect sphere and 0 to a flat surface. At day 1, the sphericities for RBCs stored with and without CPDA-1 did not show any significant difference. However, in the absence of CDPA-1, the sphericities of RBCs exhibited significant variations on day 5. After day 13, in the absence of CPDA-1, all RBCs were spherocytes. The mean sphericity values for the RBCs stored without CPDA-1 at days 1, 5, 13, 20, 27, 34, and 41 of the storage period were 0.635 ± 0.037, 0.774 ± 0.134, 0.953 ± 0.017, 0.956 ± 0.010, 0.959 ± 0.011, 0.953 ± 0.007, and 0.949 ± 0.007, respectively. In contrast, RBCs stored with CPDA-1 exhibited a prolonged transformation from discocytes to spherocytes. The mean sphericity values for the RBCs stored with CPDA-1 at the corresponding storage periods were 0.631 ± 0.065, 0.677 ± 0.107, 0.836 ± 0.132, 0.843 ± 0.094, 0.872 ± 0.135, 0.893 ± 0.072, and 0.906 ± 0.012, respectively.

### Decreased deformability of the stored RBCs

To investigate the changes in the deformability of individual RBCs over the storage period, the dynamic membrane fluctuations of the cells were quantitatively measured. Dynamic membrane fluctuations manifest the deformability of RBC membranes, which is determined by the structures of the membrane cortex and the viscosity of the cell cytoplasm^24^. The deformability of RBCs is strongly related to their ability to pass through small capillaries and can be altered due to various pathophysiological conditions^25^,^26^,^27^,^28^,^29^,^30^,^31^,^32^.

**Figure 3:**
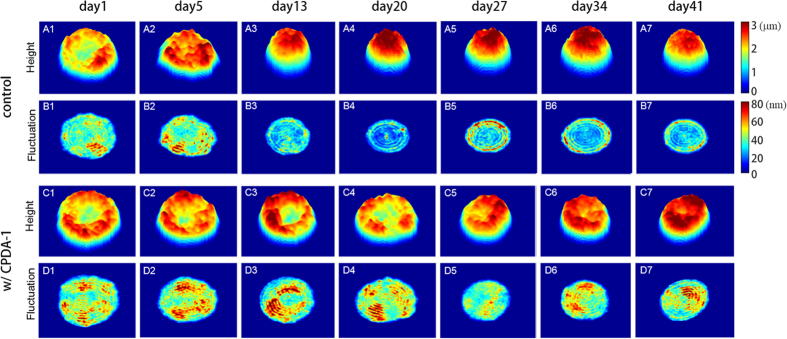
Representative 2-D topographical images of stored RBCs without CPDA-1 as the control (**A1–A7**) and with CPDA-1 (**C1–C7**) at days 1, 5, 13, 20, 27, 34, and 41 of the storage period (left to right), respectively. (**B,D**) Corresponding membrane fluctuation maps.

The dynamic membrane fluctuations in RBCs were quantitatively and precisely measured using cDOT^23^. The temporal full-field optical phase images of individual RBCs were measured with normal laser illumination, from which the mean and dynamic height maps of RBCs were obtained (see Materials and Methods). The representative mean height maps of individual RBCs at each tested storage day are presented in Fig. 3A1–A7. Consistent with the results of the 3-D rendered isosurfaces (Fig. 1), the results of the mean cell shapes show that RBCs stored without CPDA-1 underwent a fast morphological transformation from discocytes to spherical shapes; RBCs lost their characteristic biconcave shapes after day 5. In contrast, RBCs stored with CPDA-1 exhibited a significantly delayed transformation; biconcave shapes were still found at day 41 for many of the RBCs.

To quantify the deformability of individual RBCs, the membrane fluctuations were calculated by spatially averaging the root-mean-squared (RMS) height displacements over the cell. The representative RMS height displacements maps of RBCs stored without and with CPDA-1 at various storage days are shown in Fig. 3B1–B7, respectively. For RBCs stored without CPDA-1, the RMS height displacements of the spherocytes found in groups with a long storage period were significantly lower than those of the discocytes. However, the RMS height displacements of the RBCs stored with CDPA-1 were not dramatically decreased.

**Figure 4:**
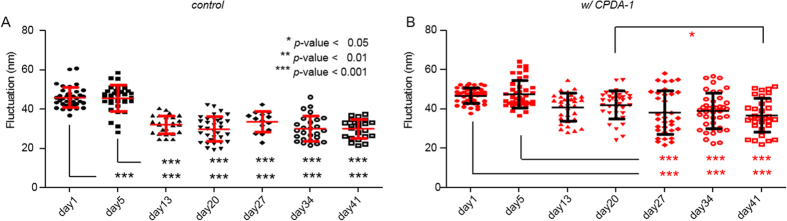
Mean membrane fluctuations of individual stored RBCs without CPDA-1 as the control (**A**) and with CPDA-1 at days 1, 5, 13, 20, 27, 34, and 41 of the storage period.

The mean membrane fluctuations of the RBCs on various days of the storage period are shown in Fig. 4. The membrane fluctuations of the RBCs stored without CPDA-1 rapidly decreased after 13 days of storage, whereas those of the RBCs stored with CPDA-1 gradually decreased and exhibited significant cell-to-cell variations. The mean values for the membrane fluctuations of RBCs stored without CPDA-1 were 46.0 ± 5.2, 45.6 ± 6.6, 32.1 ± 4.6, 30.0 ± 6.3, 33.7 ± 5.3, 30.2 ± 6.5 and 30.1 ± 4.9 nm, and in contrast with CPDA-1 with values of 46.8 ± 4.1, 47.6 ± 7.1, 41.0 ± 7.1, 42.1 ± 7.1, 38.2 ± 11.0, 39.1 ± 9.0 and 36.8 ± 8.6 nm at days 1, 5, 13, 20, 27, 34, and 41 of the storage period, respectively.

**Figure 5:**
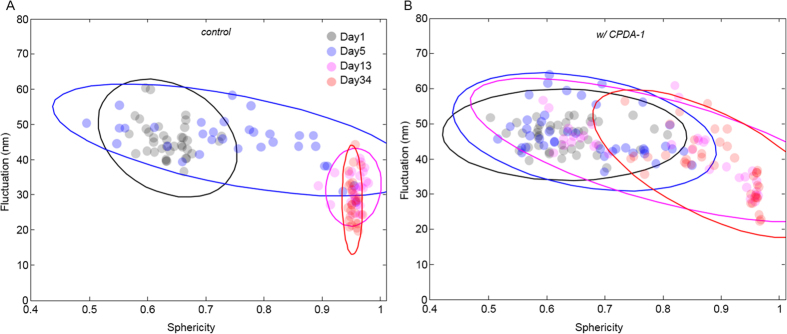
Correlation maps between sphericity and mean membrane fluctuations of (**A**) stored individual RBCs without CPDA-1 and (**B**) with CPDA-1 at days 1, 5, 13, 20, 27, 34, and 41 of the storage period.

To further investigate the morphological alterations over the storage period, a correlation analysis was done between the fluctuations and sphericity. As shown in Fig. 5, the correlations between the fluctuation levels and sphericity show similar trends for the RBCs with and without CPDA-1; the fluctuation level decreases as the sphericity increases. These correlations exhibited different alterations as the storage period increased for RBCs stored with or without CPDA-1 (see fitted 2-D Gaussian circles). By day 5 of the storage period without CPDA-1, the mean fluctuations of the RBCs gradually decreased as the sphericity increased from 0.6 to 1. After two weeks of storage, all the RBCs stored without CPDA-1 became highly spherical shapes, and their mean fluctuation levels were within 20–40 nm and significantly lower than the fluctuations of healthy discocytes on day 1. For RBCs stored with CPDA-1, the correlation clusters slowly moved toward the bottom right side in the scatter plot as the storage period increased; the clusters exhibited significant deviations compared to that of the RBCs stored without CPDA-1. Additionally, the results show that there exists an abrupt transition in a sphericity value of 0.9 regardless of the addition of CPDA-1. For RBCs with a sphericity smaller than 0.9, the fluctuation levels increased slowly as the sphericity increases at a constant rate (approx. a 20 nm decrease in fluctuations for a 0.5 increase in sphericity). However, the fluctuation levels abruptly decreased for RBCs with a sphericity higher than 0.9. This transition in the correlation implies that the substantial changes in the deformability are associated with spherocytosis. Our results clearly show that CPDA-1 prevents spherocytosis and helps RBCs maintain a high deformability.

### Hb content and concentration of stored RBCs

To further investigate alternations in the cytoplasmic biochemical properties during the storage period, the hemoglobin (Hb) content and concentration in stored RBCs with or without CPDA-1 were quantified from the measured 3-D RI maps. Because the RBC cytoplasm is mainly composed of Hb and the RI of an RBC is linearly proportional to the Hb concentration, the total Hb content of individual RBCs was retrieved from the measured 2-D optical field images of the cells^33^,^34^,^35^,^36^. Then, the Hb concentrations of individual RBCs were calculated by dividing the Hb content by the cell volume (see Materials and Methods).

**Figure 6:**
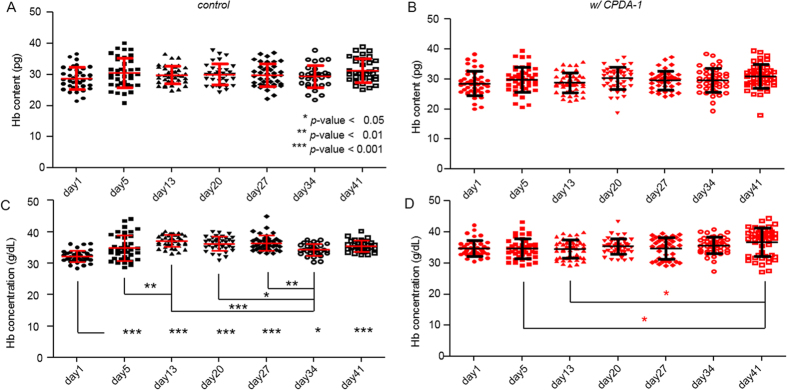
Hb content and concentration in the stored RBCs without CPDA-1 as the control (**A,C**) and with CPDA-1 (**B,D**), respectively, at days 1, 5, 13, 20, 27, 34, and 41 of the storage period.

Figure 6 shows the results for the Hb contents and concentrations. After six weeks of storage, there were no significant changes in the Hb content for both groups of RBCs stored with and without CPDA-1 (Fig. 6A,B); the values for the Hb contents were within the reference range (28.5–33.5 pg) for healthy RBCs. The mean values for the Hb content of RBCs stored without CPDA-1 were 28.8 ± 3.6, 30.5 ± 4.7, 29.9 ± 2.9, 30.0 ± 3.4, 29.8 ± 3.7, 29.4 ± 3.6 and 31.3 ± 3.8 and in contrast with CPDA-1 28.6 ± 4.1, 29.8 ± 4.3, 28.8 ± 3.3, 30.3 ± 3.8, 29.6 ± 3.2, 29.7 ± 4.0 and 30.9 ± 4.0 at days 1, 5, 13, 20, 27, 34, and 41 of the storage period, respectively. These results are consistent with a recent study using digital holographic microscopy^37^.

Contrary to the surface area, sphericity, and membrane fluctuation, the Hb concentration for both groups of RBCs did not exhibit any sudden alterations to another equilibrium state over 41 days of storage (Fig. 6B,D). Statistically, however, day-1 RBCs without CPDA-1 have Hb concentrations significantly lower than those of other days, contributed slightly but not significantly by both large volumes and low Hb contents. The mean values for the Hb concentrations of RBCs stored without CPDA-1 were 32.2 ± 1.9, 34.9 ± 4.0, 37.1 ± 1.9, 36.2 ± 2.2, 36.4 ± 2.4, 34.3 ± 1.9 and 35.4 ± 1.8 pg/dL, and with CPDA-1 were 34.8 ± 2.5, 34.6 ± 3.2, 34.5 ± 3.0, 35.4 ± 2.5, 34.7 ± 3.4, 35.7 ± 2.7 and 36.7 ± 4.4 pg/dL, at days 1, 5, 13, 20, 27, 34, and 41 of the storage period, respectively. Increases in the Hb concentration observed in the RBC group without CPDA-1 during first two weeks of storage might be affected by the dramatic morphological transformations of the RBCs such as echinocytosis or hemolysis caused by intracellular ATP depletions or disappearances of aged red cells, which can alter the experimentally measurable RBC pool. This explanation is still compatible with the reported invariance of the Hb concentration during blood storage periods in the complete blood count (CBC) test^2^ because flow cytometry based technique determines Hb content by hemolyzing all the RBCs; thus, it cannot fully reflect the hemolysis or echinocytosis that occurred. However, careful consideration should be given to whether the increase in Hb concentration in RBCs without CPDA-1 is indeed a consequence of the CPDA-1 deficiency. First, the retrieved Hb concentration of individual RBCs is more vulnerable to error measurements than other parameters because the Hb concentration is obtained from independently measured Hb content and cell volume (See Materials and Methods). At the same time, the observation that even control RBCs at day1 exhibit Hb concentrations lower than those of the same day RBCs with CPDA-1 (p-value < 0.001, obtained from simple two-tailed t-test) may imply another possibility that the measured sample in the day one control group cannot sufficiently represent the whole population. Despite these issues, all the mean values for the Hb concentrations in the two groups were still within the reference range for healthy RBCs (33–36 g/dL). This result may suggest that the cytoplasmic Hb proteins were not severely degraded or removed during the six weeks of storage, despite the dramatic alterations in the morphological and mechanical properties.

## Conclusion and Discussion

In this paper, we investigated alterations in individual human RBCs during blood storage. With 3-D QPI techniques, the morphological (cellular volume, surface area, and sphericity), biochemical (Hb content and Hb concentration), and mechanical properties (membrane fluctuation) of individual RBCs stored with and without CPDA-1 were systemically quantified.

Our optical measurements showed that the cellular volume and the cytoplasmic Hb content of stored RBCs did not change significantly up to 6 weeks of the storage time regardless of the CPDA-1 treatments. In the case of the Hb concentration, day1 RBCs without CPDA-1 had Hb concentrations considerably lower than those of RBCs in different storage periods, possibly as a result of either a slight water efflux through a membrane or altered accessible RBC pools, or both. ATP depletions and hemolysis of aged red cells accompanied by a lack of CPDA-1 along storage period might account for these alterations in RBC characteristics.

However, the most notable alterations in the stored blood can be found in its surface area and sphericity. The surface areas of the RBCs significantly decreased during the first two weeks with sphericity values of unity. The morphologies of the RBCs dramatically transformed in less than one week of storage. In the absence of CPDA-1, 60% of the RBCs stored without CPDA-1 were a non-discocyte shape at day 5; after day 13, all the RBCs became spherical shapes. These results – the decrease in cell membrane area and the increase in sphericity – imply that vesiculation induces spherocytosis in the stored RBCs. This storage-induced spherocytosis seems to cause a significant decrease in cell deformability; membrane fluctuations in the RBCs decreased by 53% after two weeks of storage.

In the RBCs stored in the presence of CPDA-1, the morphological transformation to spherocytosis was significantly delayed compared to the RBCs stored without CPDA-1. In the presence of CPDA-1, 80% of the RBCs remained as discocytes at day 5. Furthermore, there was a significant fraction of discocytes after six weeks of storage (38% of the RBCs had a sphericity less than 0.9), although the number of sphere-like RBCs gradually increased as the storage period increased. Thus, despite the previous work reported that RBCs maintained a discocyte shape during six weeks of storage^14^, it does not contradict our result when considering that they used an apheresis technique and the banked blood of donors in the preservation solution. Their banked blood can be understood as an opposite counterpart of our stored RBCs without CPDA-1. The decreasing trend in cell deformability is qualitatively consistent with a previous report^3^, in which the averaged values of the deformation index of the RBCs were significantly reduced after two weeks of storage (approx. 39%).

It is well known that CPDA-1 extends the survival days of stored RBCs by providing adenine for maintaining cytoplasmic ATP levels^1^,^38^. Our results clearly show that distinct trends in stored RBCs - the morphological transformation from discocytes to spherocytes as well as a decrease in membrane deformability – were considerably reduced in the presence of CPDA-1 at the individual cell level. This result agrees with a previous work using a narrow microchannel mimicking microcirculation^39^. In particular, the decreased amplitudes in the dynamic membrane fluctuation decreases over the storage time (Fig. 4A,B) are consistent with those of previous work in which cytoplasmic ATP was depleted chemically and metabolically^25^,^40^,^41^,^42^. More importantly, our results suggest the presence of sub-groups in RBCs exhibiting various responses to CPDA-1. For example, the sphericities and dynamic membrane fluctuations after two weeks of storage showed highly dispersed values ranging from healthy levels to severe spherocytes. This significant cell-to-cell variation in the responses to CPDA-1 could be from various factors, including the different ages of the individual RBCs or various cellular levels of potassium and 2,3-DPG in the RBCs^4^.

Our results suggest measurements of sphericity can be exploited to address the expected duration of RBCs in the clinic. Traditional CBC measurements do not provide information about sphericity or cell surface area. Hence, it remains challenging to expect a survival duration of RBCs in stored blood. Currently, the reticulocyte count can be used to address the fraction of young RBCs. However, the fraction of old RBCs in the blood cannot be measured. In the view of clinical hematology, the results presented in this work may suggest the possibility that sphericity or surface area is somehow correlated with the viability of RBCs and can also be used as a proxy for the cell age of RBCs, even taking into account the fact that our experimental conditions for stored blood are not exactly same as in clinical conditions required for banked blood for transfusions (See Materials and Methods). In other words, measurements of cell sphericity may provide useful information about the expected survival duration of RBCs. In particular, a sphericity higher than 0.9 or membrane fluctuations of 35–40 nm or below could be used as criteria to identify inviable RBCs. At this stage, however, we cannot tell whether the observed alterations in morphologies of the stored RBCs are indeed related to the mortality of patients in clinical transfusion. Studies on these correlations are beyond the scope of our study.

In this paper, we presented the optical measurements for various parameters of stored RBCs and investigated the progressive alterations of these retrieved parameters. We believe the present method can be usefully exploited in clinical applications for hematology in the future. For example, the present method can be used to assess non-invasive blood preservatives or the functionalities of stored blood. Using the recently developed quantitative phase imaging units^43^,^44^, existing optical microscopes in a laboratory can be converted into quantitative phase microscopy, which can extend the applicability of the present method. Towards routine clinical applications, however, further developments should be made, particularly in high-throughput measurement capabilities and data analysis and management techniques.

## Materials and Methods

### Ethics statements and Blood sample preparation

Human blood studies were done according to the principles of the Declaration of Helsinki and approved by the responsible ethics committee of Eulji University Hospital (IRB project number: 2012-0128, Daejeon, Republic of Korea). All experimental protocols were approved by the institutional review board of KAIST (KH2013-22). The participant provided written informed consent to participate in this study which was approved by the ethics committees and IRB.

Blood samples from a healthy individual 20 years of age or older were obtained through a regular course of patient care after approval of the procedures by the IRB for the remaining blood. First, blood of 4 mL was collected from a donor and put into an EDTA-treated anticoagulant tube and then divided into two tubes. In one of the tubes, CPDA-1(citrate-phosphate-dextrose with adenine, C4431, Sigma-Aldrich, U.S.A.) was added to whole blood at a 14:100 v/v ratio. The other tube without CPDA-1 (control group) was kept as the control sample. At this stage, it needs to be noted that our experimental conditions for the CPDA-1 group are not exactly same with those of clinically used banked blood, in which the apheresis technique is usually used, and EDTA anticoagulant tube is not used. Although, because it has been widely accepted that EDTA hardly affects RBC morphology, EDTA tubes were initially chosen as our blood containers for the experimental purpose to address the effects of CPDA-1 on the RBC properties during blood storage. To validate our approach, we confirmed that the results obtained from using the citrate tube were statistically same with those using the EDTA tube during three weeks of blood storage (see Supplementary Fig. S1). Whole blood in the control and the CPDA-1 groups were stored at 4 °C throughout the storage period of 41 days and agitated occasionally. Every few days up to 41 days, blood from each tube was tested for a quality check using 3-D optical tomography. Before measurements, small aliquots of stored whole bloods in the anticoagulant tubes were taken out and then diluted 300 times in Dulbecco’s PBS (Gibco®, New York, U.S.A.). The main purpose of this dilution is to separate individual RBCs such that measurements of RBCs are not affected by adjacent cells. Then, diluted blood solutions were squeezed between top and bottom coverslips and loaded on a sample stage of an inverted microscope.

### Statistical Analysis

Throughout the manuscript, all retrieved RBC parameters were presented as the mean ± standard deviation. To compare the mean values of the RBC parameters with different storage durations for each RBC group, we used one-factor analysis of variance (ANOVA). As a posthoc analysis, obtained *p*-values were adjusted with the Bonferroni method to make the family-wise error rate, which denotes the possibility of rejecting one or more true null hypotheses in all performed pairwise comparisons, less than 0.05. Accordingly, *p*-value for statistical difference was set to be 0.05.

### Common-path diffraction optical tomography (cDOT)

Optical tomography is a technique that can measure the 3-D distribution of RI of individual RBCs. As an illumination source, a diode-pumped solid state laser (*λ* = 532 nm, 50 mW, Cobolt, Solna, Sweden) was used. The sample, diluted blood sandwiched between two cover glasses of 25 × 50 mm (C025501, MATSUNAMI GLASS Ind., LTD., JAPAN) is placed between the condenser lens (UPLSAPO 60×, numerical aperture (N.A.) = 0.9, Olympus, Japan) and objective lens (UPLSAPO 60× , N.A. = 1.42, Olympus, Japan).

For 3-D RI tomography, the optical fields obtained with various incident illumination angles were measured by using common-path laser-interferometric microscopy^23^,^45^. By rotating a two-axis galvanometric mirror (GVS012/M, Thorlabs, USA), the incident angles of the illumination beams to a sample are controlled. The second galvanometer mirror reflected the beam from a sample to have the same optical path regardless of the incident illumination angles.

Holograms of the sample were recorded using the principle of common-path interferometry^46^,^47^. After the second galvanometer mirror, a diffraction grating (92 grooves mm^−1^, #46-072, Edmund Optics Inc., NJ, U.S.A.) spatially split the scattering beams and then, spatially filtered the 0^th^ order beam as the reference interfered with the 1^st^ order beam as a sample beam. Then, the interferograms were recorded on a high-speed sCMOS camera (Neo sCMOS, ANDOR Inc., Northern Ireland, UK) while the incident beam was scanning spirally with 300 different angles. The total magnification was 240 by an additional 4-*f* system. Then, optical field images, containing both the amplitude and phase maps, of the sample were retrieved using a phase retrieval algorithm^48^. From the retrieved optical fields, the 3-D RI distribution of the sample was reconstructed using the optical diffraction tomography algorithm, found elsewhere^23^.

Alternatively, a recently introduced commercial quantitative phase microscopy, based on a digital micro-mirror device^49^,^50^, can also be used to measure both the 3-D RI tomography and dynamic membrane fluctuations of RBCs.

### Analysis of the red cell parameters

From the measured 3D RI tomograms and the 2D dynamic membrane fluctuations, six red cell parameters were retrieved, including morphological (cell volume, surface area and sphericity), chemical (Hb content and Hb concentration), and mechanical (membrane fluctuation) parameters. To measure the morphological parameters, we used the reconstructed 3-D RI maps^51^. The whole volume of an RBC was calculated by integrating all voxels inside individual RBCs. The space corresponding to the cytoplasm of an RBC was selected by RI with a higher value than the threshold. The threshold was defined by 50% of the RI difference between the maximum RI of the cell *n*_*cell_max*_ and the surrounding medium *n*_*m*_ for determining the cell boundary, i.e., *n*_*thresh*_ = *n*_*m*_ + 0.5 · (*n*_*cell_max*_ − *n*_*m*_). Then, the total number of voxels was multiplied by the magnification of the optical system to translate to a length scale. Next, for the surface area measurements, the isosurfaces of individual RBCs were reconstructed from the volume data of the 3-D RI maps. The surface area of the isosurface was measured by the sum of the areas of all the patch faces, which were broken down into small triangular pieces. In addition, the sphericity *SI*, a dimensionless quantity ranging from 0 to 1, was obtained as follows: *SI* = π^1/3^(6*V*)^2/3^/*A* where *V* is the volume and *A* is the surface area^51^,^52^,^53^.

To obtain the Hb content of individual RBCs, the measured 2-D phase at the normal angle was used. The Hb content of an RBC was obtained from integrating the 2-D optical phase over the entire cell area and with the RI increased by the Hb proteins, given as follows:


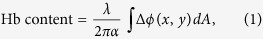


where *λ* is the wavelength of the illumination laser light (532 nm); *α* is the RI increment (0.2 mL/g)^35^,^36^, and Δ*ϕ*(*x,y*) is a 2-D optical phase. In addition, the Hb concentration in an RBC was obtained from the Hb content divided by the cellular volume^54^,^55^.

The dynamic membrane fluctuations in RBCs can be quantitatively and precisely measured using cDOT^23^,^24^. Dynamic full-field optical phase images of a RBC *Δϕ*(*x*, *y*, *t*) can be measured with a normal-angle laser illumination, from which dynamic height maps of the RBC can be calculated as *h*(*x*, *y*, *t*) = [*λ*/(2π·Δ*n*)]*Δϕ*(*x*, *y*, *t*), and *Δn* = 〈*n*(*x*, *y*, *z*)〉 − *n*_*m*_ is the difference between the mean RI of the RBC cytoplasm 〈*n*(*x*, *y*, *z*)〉 and the surrounding buffer medium *n*_*m*_.

The representative cell height images of individual cells in each group are calculated as the temporally averaged cell heights, *h*_*m*_(*x*, *y*) = 〈*h*(*x*, *y*, *t*)〉, and are presented in Fig. 3A,C. To measure the mechanical parameter, we calculated the dynamic membrane fluctuation from the successively measured instantaneous height map *h*(*x*, *y*, *t*) given as:


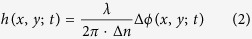


The values for the membrane fluctuations were calculated by averaging the root-mean-square of the height displacement over the cell area as follows:


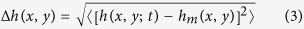


where *h*_*m*_ is the time averaged height at the cell surface^56^. To preclude erroneously measured membrane fluctuations of individual RBCs into our result, RBCs either in translational motion or abnormally attached to the bottom coverslip were excluded in the statistical analysis. Detailed analysis can also be found in previous relevant studies on malaria^57^, ethanol effect^58^, and cord blood^59^.

## Additional Information

**How to cite this article**: Park, H. J. *et al*. Measuring cell surface area and deformability of individual human red blood cells over blood storage using quantitative phase imaging. *Sci. Rep.* **6**, 34257; doi: 10.1038/srep34257 (2016).

## Supplementary Material

Supplementary Information
