# Supplementary material for: Measuring cell surface area and deformability of individual human red blood cells over blood storage using quantitative phase imaging
Source: Sci Rep. 2016 Oct 4;6:34257. doi: 10.1038/srep34257 (PMC5048416; doi:10.1038/srep34257)
Supplement: Supplementary Information [file srep34257-s1.pdf]

Measuring cell surface area and deformability of individual human red blood cells over blood storage using quantitative phase imaging

HyunJoo Park[1,¶], SangYun Lee[1,¶], Misook Ji[2,¶], Kyoohyun Kim[1], YongHak Son[2,*], Seongsoo Jang[3,*] and YongKeun Park[1,4*]

[1] Department of Physics, Korea Advanced Institute of Science and Technology, Daejeon 34141, Republic of Korea.
[2] Department of Laboratory Medicine, Eulji University Hospital, Daejeon 35233, Republic of Korea.
[3] Department of Laboratory Medicine, University of Ulsan, College of Medicine and Asan Medical Center, Seoul 05535, Republic of Korea.
[4] TOMOCUBE, Daejeon 34051, Republic of Korea

Supplementary Information

Morphological alterations of individual RBCs stored in an EDTA and citrate tube

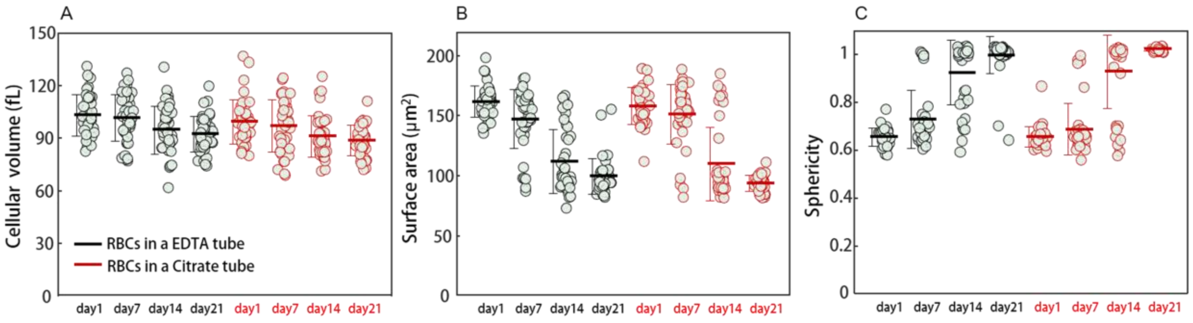

Fig. S1 Cellular volume (A), surface area (B), and sphericity (C) of stored RBCs in an EDTA tube and those in a citrate tube. Each circle denotes individual red cell measurement. Horizontal thick lines and vertical error bars respectively indicate mean values with sample standard deviations.
